# Reduced Visual‐Cortex Reorganization Before and After Cochlear Implantation Relates to Better Speech Recognition Ability

**DOI:** 10.1002/jnr.70042

**Published:** 2025-05-08

**Authors:** Anna Weglage, Natalie Layer, Jan‐Ole Radecke, Hartmut Meister, Verena Müller, Ruth Lang‐Roth, Martin Walger, Pascale Sandmann

**Affiliations:** ^1^ Cochlear Implant Centre, Department of Otorhinolaryngology, Head and Neck Surgery, Audiology and Pediatric Audiology, Faculty of Medicine and University Hospital Cologne University of Cologne Cologne Germany; ^2^ Department of Psychiatry and Psychotherapy University of Lübeck Lubeck Germany; ^3^ Centre of Brain, Behaviour and Metabolism (CBBM) University of Lübeck Lubeck Germany; ^4^ Jean‐Uhrmacher‐Institute for Clinical ENT Research University of Cologne Cologne Germany; ^5^ Department of Otolaryngology, Head and Neck Surgery Carl von Ossietzky University of Oldenburg Oldenburg Germany; ^6^ Research Center Neurosensory Science University of Oldenburg Oldenburg Germany; ^7^ Cluster of Excellence “Hearing4all” University of Oldenburg Oldenburg Germany

**Keywords:** allocation of attention, alpha power, cortical reorganization, visual face processing

## Abstract

Although a cochlear implant (CI) can partially restore auditory function, CI recipients show alterations not only in auditory but also in visual cortical processing. Yet, it is not well understood how these visual changes relate to the CI outcome and to what extent these changes are induced by auditory deprivation and the limited CI input, respectively. Here, we present a prospective longitudinal electroencephalography study which examined the deprivation‐ and CI‐induced alterations on cortical face processing by comparing visual evoked potentials (VEP) in CI users before and 6 months after implantation. A group of normal‐hearing (NH) listeners served as a control. The participants performed a word‐identification task and a face‐categorization task to study the cortical processing of static and articulating faces in attended and unattended conditions. The CI candidates and CI users showed a reduced visual‐cortex activation, a stronger functional connectivity between the visual and auditory cortex, and a reduced attention effect in the (extended) alpha frequency range (8–18 Hz) when compared to NH listeners. There was a positive correlation between the P1 VEP amplitude recorded before implantation and the speech recognition ability after implantation. Our results suggest that the CI users' alterations in cortical face processing are mainly induced by auditory deprivation and not by CI experience. Importantly, these deprivation‐induced changes seem to be related to the CI outcome. Our results suggest that the visual P1 amplitude as recorded *before* implantation provides an objective index of cortical visual reorganization that may help predict the CI outcome.

## Graphical abstract

Using two different EEG tasks with static and articulating faces, deprivation‐induced cortical changes in postlingually deafened individuals were found by means of reduced visual cortex activation and a relationship between these alterations and the CI outcome after 6 months. These alterations are not significantly affected by CI experience.

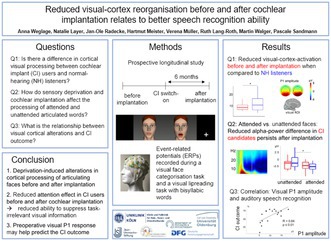

**Summary**

- Individuals suffering from any hearing impairment are usually more sensitive to the visual surrounding and, most importantly, the lip movement of any conversational partner.
- Therefore, they are able to compensate for the missing auditory input by lipreading. Even though a cochlear implant (CI) can restore the hearing ability, the input is still limited.
- Our study aims to explain the underlying cortical alterations of the visual processing in hearing‐impaired individuals receiving a CI as compared to normal hearing (NH) individuals as well as the effect of attention directed to or revoked from the lip movement.

## 1 Introduction

Severe to profound hearing loss can have drastic effects on a person's daily routine and quality of life (for review see Nordvik et al. 2018). Due to the limited auditory input, affected individuals rely on the intact modalities and compensatory strategies (Bavelier et al. 2006). The visual system, in particular, is critical because it helps with orientation, environmental change detection, and speech recognition through lipreading. Previously, several studies have reported enhanced visual abilities in congenitally deaf individuals, which, however, seem to be stimulus‐ and task‐selective (Hauthal et al. 2013). Specifically, these individuals show larger visual fields (Buckley et al. 2010; Codina et al. 2011; Stevens and Neville 2006) and faster reaction times in visual detection (Bottari et al. 2010; Chen et al. 2006; Hong Lore and Song 1991) and visuo‐spatial localization tasks (Dye et al. 2009). It seems that this facilitated visual information processing is especially pronounced in the peripheral visual field (e.g., Hong Lore and Song 1991; but see Bottari et al. 2010).

Previous studies using electrophysiology and neuroimaging techniques have reported that the visual improvements in deaf individuals are accompanied by cortical reorganization (Lomber et al. 2010; Bavelier et al. 2006), as reflected by functional changes within the visual cortex (i.e., intra‐modal plasticity) and/or by take‐over of the deprived auditory regions by the remaining sensory systems (i.e., cross‐modal plasticity) (for reviews, see Stropahl et al. 2017 and Kral and Sharma 2023). Specifically, congenitally deaf individuals, when tested with visual stimuli, recruit not only the visual but also the auditory cortex (Finney et al. 2001). Similarly, cross‐modal activation in the auditory cortex has been reported in individuals with postlingual onset of hearing loss, which seems to develop within a few months of auditory deprivation (Campbell and Sharma 2014). There is also increasing evidence of cross‐modal cortical changes in postlingually deafened individuals who use a cochlear implant (CI) (Sandmann et al. 2012; Rouger et al. 2012).

The CI is a bionic device that can be implanted in individuals with profound sensorineural hearing loss and who do not sufficiently benefit from conventional hearing aids. The CI can partially restore the auditory function by direct electrical stimulation of the auditory nerve (Zeng 2011 ). However, unlike natural acoustic hearing, the CI's electrical signals are limited in spectral and temporal information (Drennan and Rubinstein 2008). It is therefore not surprising that the speech recognition ability with a CI, as referred to as the CI outcome in clinical context (Hoppe et al. 2019), is limited. Nevertheless, the CI outcome is known to be affected by various individual factors such as the age at implantation or the time of deafness before implantation (Green et al. 2005; Lazard et al. 2012). Specifically, deprivation‐induced cortical reorganization is thought to affect the CI outcome, although the adaptiveness or maladaptiveness of this plasticity prior to implantation has been debated (Heimler et al. 2014; Paul et al. 2022). Besides, the novel auditory experience via the CI may induce additional cortical changes (Ito et al. 2004; Sandmann et al. 2015). Given the low number of prospective longitudinal studies, it is currently not well understood whether the cortical reorganization induced by sensory deprivation persists, reverses, or even proceeds after implantation (Stropahl et al. 2017; Rouger et al. 2012). A better knowledge of cortical alterations before and after implantation, therefore, is clinically relevant, as cortical measures that indicate the degree of cortical reorganization may be helpful to predict the CI outcome already at the time before implantation (Anderson et al. 2019; Rouger et al. 2012; Strelnikov et al. 2013).

Several previous studies have examined the processing of faces in congenitally deaf individuals and in CI users. It has been shown that congenitally deaf individuals rely on visual information during face‐to‐face communication to compensate for the missing auditory input (Kral 2013; Mitchell et al. 2013; Woodhouse et al. 2009). Compensatory processes have also been reported for CI users, showing superior lipreading abilities before and after implantation (Rouger et al. 2007; Stropahl et al. 2015; Anderson et al. 2019) and pointing to a functionally specialized pattern of cortical face processing (Stropahl et al. 2017). Specifically, Stropahl et al. (2017) reported alterations in the N170 evoked potential, which is elicited approximately 170 ms after a face stimulus onset (Bentin et al. 1996; Bötzel and Grüsser 1989; Rossion and Jacques 2008) and which originates from the fusiform area (Haxby et al. 2000; Kanwisher et al. 1997; Kanwisher and Yovel 2006). Interestingly, the N170 response to faces was enhanced in CI users when compared to normal‐hearing (NH) listeners, suggesting experience‐related alterations in cortical visual face processing in these individuals (Stropahl et al. 2015).

Electroencephalography (EEG) represents an interesting tool for studying cortical plasticity in congenitally deaf individuals (Bottari et al. 2011; Hauthal et al. 2014) and in postlingually deafened CI users (Sandmann et al. 2009, 2015; Sharma et al. 2002; Viola et al. 2012). Visual evoked potentials (VEPs) have a high temporal resolution, allowing researchers to track single steps of the cortical processing (Biasiucci et al. 2019; Michel and Murray 2012). Time‐frequency analysis of the EEG data provides additional, i.e., frequency‐related information about the neural processes involved in visual perception. For instance, neural activity in the alpha frequency range (8–12 Hz) varies as a function of the level of attention (Berger 1929; Adrian and Matthews 1934) and is affected by anticipation of upcoming stimuli (Andersen et al. 2008; Zhang and Luck 2009), as reflected by a decrease in alpha power in sensory regions that process upcoming targets. This observation converges with the generally accepted view that cortical processing in the primary sensory cortex is influenced not only by bottom‐up sensory inputs but also by top‐down task‐dependent processes, such as the attentional state (Polley et al. 2006). Regarding CI users, however, it remains to be clarified whether these individuals show experience‐related alterations in alpha power during the processing of attended and unattended articulating faces, which would point to changes in the allocation of attention during visual processing. Therefore, employing an alpha power analysis for VEPs recorded in conditions with different static faces on the one hand and attended and unattended articulating faces on the other hand seems to be a promising approach for a differentiated understanding of cortical alterations in CI users. Indeed, a first insight into the attentional processing of articulating lips has been given by Paul et al. (2022) who found a group‐specific pattern of increase and decrease in alpha power for the CI users when compared to NH listeners.

Here, we present a prospective longitudinal EEG study which examined CI users before and after cochlear implantation and a group of NH controls by means of a word‐identification task and a face‐categorization task. As far as we are aware, this is the first study to systematically analyze deprivation‐ and CI‐induced effects on cortical processing of static and articulating faces in attended and unattended conditions. In contrast to previous studies, which focused on more simple stimuli such as dot patterns (Hauthal et al. 2013) and chequerboards (Sandmann et al. 2012), the present study used more complex, articulating face stimuli that were produced by a computer animation of a talking head (Fagel and Clemens 2004; Schreitmüller et al. 2018). Importantly, the articulated words were physically identical in the two tasks, but they differed in terms of behavioral relevance, as these words were attended in the word‐identification task (Target stimuli), and they were ignored in the face‐categorization task (NonTarget stimuli). In sum, the use of different groups, recording sessions, and conditions allowed us to address the following research questions:

1. Is there a difference in the cortical (sensory) processing of static and articulating faces between postlingually deafened individuals (before implantation) and NH listeners?
2. Do postlingually deafened individuals (before implantation) and NH listeners differ in their allocation of attention to static and articulating faces?
3. Does CI experience affect the cortical (sensory) processing and allocation of attention in visual conditions with static and articulating faces?
4. How do visual cortical alterations in CI users relate to the CI outcome, as measured by an auditory monosyllabic word test?

Based on previous results from congenitally deaf individuals and CI users (Hauthal et al. 2014; Stropahl et al. 2017), we expected deprivation‐ and CI‐induced alterations in cortical face processing. We also expected a relationship between cortical visual activation and auditory speech recognition ability, which would support previous reports of a link between cortical reorganization and the CI outcome (Sandmann et al. 2012; Strelnikov et al. 2013). Overall, our results are of clinical relevance. Given that cortical reorganization has been identified as one of several factors that contribute to the variability in CI outcome (Lazard et al. 2012), it is important to better understand whether VEPs provide an objective index of intra‐modal cortical reorganization which might help predict the speech recognition ability with the CI after implantation.

## 2 Materials and Methods

### 2.1 Participants

The total number of adult volunteers in this study was 37, of which 20 suffered from profound to severe hearing loss and were supplied with a CI during the time of the study. The first of two experimental sessions took place prior to implantation (29.65 ± 34.22 days), and the second measurement occurred approximately 6 months (5.71 ± 0.47 months) after the initial fitting of the sound processor. Three of the participants did not continue study participation after the first measurement and thus were excluded from the analysis. One CI candidate withdrew from the implantation and the other two were not able to attend the second measurement due to personal reasons. The resulting 17 hearing impaired participants (9 females, 8 males) had a pure tone average (PTA) of 99.91 ± 16.42 dB HL, and their age ranged from 36 to 74 years (mean 56 ± 11.53 years) at the first measurement. Seventeen age and gender matched normal hearing (NH) controls participated as a control group (mean age: 57.95 ± 13.53 years). All participants had normal or corrected‐to‐normal visual acuity according to the Landolt test (Landolt C; Wesemann et al. 2010) and were German native speakers. Participants reported no history of mental illness, and Beck depression inventory was unobtrusive (Beck et al. 1961). Among the 17 cochlear implant candidates, two individuals had already been implanted on the contralateral side, 13 individuals used a hearing aid (HA) on the contralateral side, and 2 individuals were unaided (see Table 1). On average, the duration of deafness was 24.71 ± 18.13 years, and all participants had a postlingual onset of hearing loss. The ‘age at onset of profound hearing loss’ (45.28 ± 15.97 years) refers to the age at which the hearing loss became too severe to be treated with conventional hearing aids and the ‘duration of deafness’ was calculated as the time between the ‘onset of profound hearing loss’ and the experiment date. To verify age‐appropriate cognitive abilities, the DemTect Ear test battery was used (Brünecke et al. 2018), an adjusted version of the conventional DemTect (Kalbe et al. 2004), especially developed for patients with hearing disabilities. Speech recognition abilities 6 months after the initial fitting of the speech processor were measured using the German Freiburg monosyllabic word test (Hahlbrock and Zöllner 1970) at a sound intensity level of 65 dB SPL.

**TABLE 1:** Demographic information of the CI participants (AB = advanced bionics, AS/AN = auditory synaptopathy/auditory neuropathy, HA = hearing aid, HL = hearing loss, PTA = pure tone average). The monosyllabic word test score was measured 6 months after implantation.

Age (years)	Gender	CI side	Etiology	Duration HL (years)	PTA [dB HL] (CI ear, pre implantation)	Other ear	CI manufacturer	Monosyllabic word tes (%)
44	f	Right	Progredient	12	94	HA	Cochlear	75
56	m	Left	Hereditary	43	111.5	CI	MedEl	75
64	m	Left	Sudden deafness	31	84.25	HA	AB	80
70	f	Right	Hereditary	4	118	HA	MedEl	75
68	m	Left	Sudden deafness	19	77.25	HA	Cochlear	60
46	f	Right	AS/AN	16	71.75	HA	Cochlear	80
75	m	Right	Unknown	4	107.25	HA	MedEl	65
39	f	Left	Unknown	32	100.75	HA	Cochlear	75
63	m	Left	Progredient	53	84	HA	MedEl	30
36	m	Right	Progredient	32	91.25	HA	Cochlear	45
74	f	Left	Unknown	12	96.25	CI	AB	75
57	f	Right	Sudden deafness	1	135	—	AB	65
66	m	Left	Unknown	21	98	HA	AB	60
56	f	Left	Sudden deafness	6	94.25	HA	MedEl	80
59	f	Right	Hereditary	59	107.75	HA	Cochlear	70
54	f	Right	Unknown	49	122.75	—	Cochlear	10
59	m	Left	Unknown	26	104.5	HA	Cochlear	45

All participants gave written informed consent in line with the Code of Ethics of the World Medical Association (Declaration of Helsinki, 2013). The study was approved by the Ethics Commission of Cologne University's Faculty of Medicine (application number 18‐197).

### 2.2 Behavioral Lipreading Task With Natural Speakers

Like previous studies (Stropahl et al. 2015; Stropahl and Debener 2017; Layer et al. 2022), we measured the lipreading ability by means of a behavioral lipreading test, using three natural speakers (see Stropahl et al. 2015) who articulated 21 different monosyllabic words from the German Freiburg monosyllabic word test (Hahlbrock and Zöllner 1970). These words were presented on a computer screen in front of the participants. The participants were asked to verbally report the word they understood after each muted video.

### 2.3 Stimuli and Procedure for the EEG Paradigms

**FIGURE 1:**
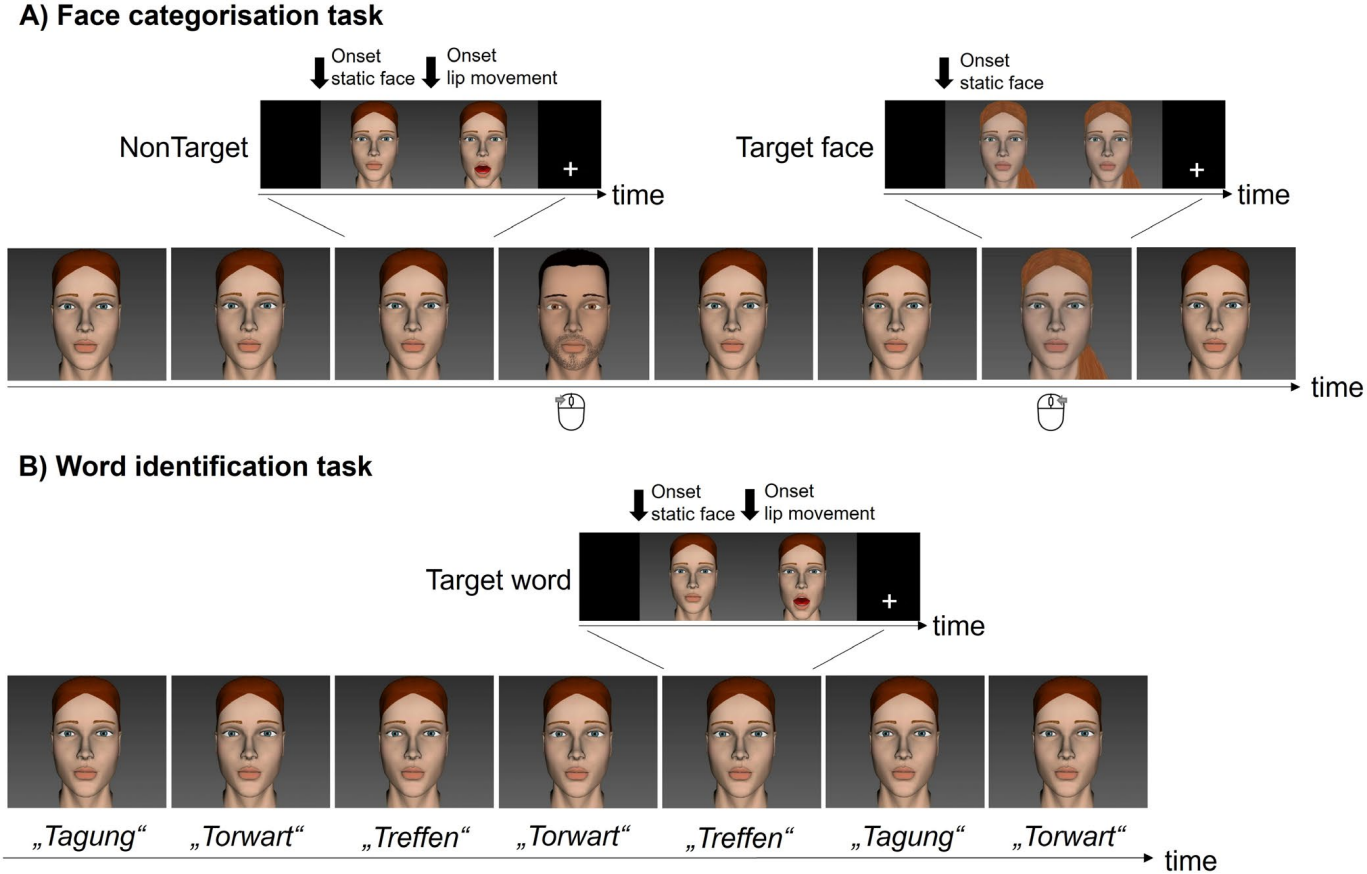
(A) Depiction of the face categorization task with frequent NonTarget stimuli and infrequent Target face stimuli. The Target face stimuli were either clearly male or female and had to be assigned by button press. (B) Depiction of the (purely visual) word identification task with Target word stimuli consisting of the three German words *Tagung (conference), Torwart (goalkeeper)*, and *Treffen (meeting)*. Note that the physically identical articulated words were presented once in the face categorization task in an unattended condition (NonTarget) and once in the word identification task in an attended condition (Target word). Also note that all face stimuli started with a static face. A subsequent lip movement (after 500 ms) was present only in the NonTargets (face categorization task) and the Target words (word identification task).

The purely visual stimuli for the two EEG paradigms consisted of videos produced by ‘The Modular Audiovisual Speech Synthesizer’ (MASSY; Fagel and Clemens 2004), a computer‐based video animation of a talking head. A talking head was used as it allows creating highly controlled stimuli tailored to the different tasks described below. The animation either articulated one of three different German words (‘*Tagung’* [ˈtaːɡʊŋ] *(conference), ‘Torwart’* [ˈtoːɐ̯vart] *(goalkeeper), and ‘Treffen’* [ˈtrɛfn] *(meeting)*) or showed a static face, whereby the face was adapted to be either clearly male or female (Figure 1). The three words were selected in advance so that the distinction of the lip movement is well possible by the time of the first vowel, without the initial lip movement (first letter) already allowing a decision. All stimuli were preceded by a blank screen for 500 ms, which was followed by the respective video (duration: 1660 ms). The video started with a static face for 500 ms in each condition before the onset of the lip movement. The trials ended with a fixation cross for no longer than 1500 ms. The fixation cross disappeared by a button press of the participants.

The participants were seated in front of a screen in a dimly lit and sound‐attenuated room. The stimuli were presented using the Presentation software (Neurobehavioral Systems, version 21.1) on a 68 cm wide screen at a viewing distance of 160 cm. Two different paradigms were completed during each experimental session: A face categorization task (Figure 1A) and a word identification task (Figure 1B). Regarding the *face categorization task*, the presented video sequence comprised 402 stimuli (321 NonTarget stimuli consisting of a static face and a lip movement, and 81 Target face stimuli consisting of different static faces) which were presented in a pseudorandomized order. This task, which can also be referred to as a visual oddball paradigm, required the participants to detect the infrequent Target face stimuli while ignoring a series of NonTarget stimuli (20% probability of Target stimuli). A Target face stimulus was at least followed by three NonTarget stimuli. Participants were instructed to ignore the NonTarget stimuli and to categorize the Target face stimuli into male or female by an assigned button press (Figure 1A). This paradigm allowed for analyzing the cortical response not only to the onset of the Target face stimuli but also to the lip movements of the ignored, that is, unattended NonTargets. The experiment was divided into three blocks of 7 min each, resulting in a total of 21 min recording time.

In the second task (*word identification task*), the participants were asked to recognize one of the three different words as a Target word and to press an assigned button for the Target word and another one for the NonTargets (Figure 1B). In contrast to the first task, this paradigm required the participants to focus their attention on the lip movements of the talking head; that is, the words were processed in an attended condition. The second experiment was also divided into three blocks with a total number of 243 stimuli (81 per word, each word was the Target word in one block), presented in a pseudorandomised order. Each trial had an approximate length of 3 s, resulting in a total time of 12 min for this paradigm. Note that the articulated words presented in the word identification task were physically identical to the NonTarget stimuli in the face categorization task, with the difference that the words (i.e., lip movements) were processed in an attended condition, while in the face categorization task, the NonTarget stimuli (i.e., lip movements) were processed in an unattended condition. Both paradigms were practised in advance to allow the subjects to become acquainted with the different faces and the lip movements they had to recognize.

### 2.4 Data Recording and Analysis

EEG data were continuously recorded by 61 Ag/AgCl actiCAP slim active electrodes (EasyCap) placed across the head according to the extended 10/20 system. Two additional electrodes were placed on the outer canthi and under the left eye to record electro‐oculograms. A reference electrode was placed at the tip of the nose, and the ground electrode was placed on the midline, anterior to AFz. Two BrainAmp DC amplifiers (Brainproducts, http://www.brainproducts.de) were used in the AC coupled mode with a time constant of 10 s to record the data; the sampling rate was 1000 Hz, and electrode impedances were kept below 10 kΩ.

#### 2.4.1 Behavioral Data

In a first step, all trials with missing or false alarm responses were removed from the dataset. The mean hit rate and the reaction time, as defined by a response in the time range between 200 and 2000 ms after stimulus onset, were calculated for the deviant stimuli of the face categorization task and the stimuli of the word identification task.

#### 2.4.2 EEG Preprocessing

Data was analyzed with EEGLAB (Delorme and Makeig 2004) for the MATLAB environment (R2020a; Mathworks). The data were downsampled to 500 Hz and filtered offline using a FIR filter. The high pass half amplitude cut‐off frequency was 0.1 Hz with a transition bandwidth of 0.2 Hz, and the low pass cut‐off frequency was 40 Hz with a transition bandwidth of 2 Hz (Kaiser‐window, beta = 5.653, max. stopband attenuation = −60 dB, max. passband deviation = 0.001) (Widmann et al. 2015). At the second measurement time point, the channels located over the scalp region of the sound processor and transmitter coil were removed for the CI users (2.82 ± 0.63). An independent component analysis (ICA) (Bell and Sejnowski 1995) was computed on additionally bandpass filtered (1 Hz–40 Hz) dummy segments with a duration of 2 s, to identify and remove components assigned to ocular artifacts and other sources of stereotypical, non‐cerebral activity (Jung et al. 2000). Additionally, following the procedure of prior studies with CI users (Debener et al. 2008; Sandmann et al. 2009; Viola et al. 2012), independent components related to the electrical artifact of the CI were also identified and removed. The remaining ICA weights were then applied to the raw data filtered between 0.5 and 40 Hz. Previously removed channels in the CI users were interpolated by using a spline interpolation (Perrin et al. 1989), which enables a good localization of the dipole sources (Debener et al. 2008; Sandmann et al. 2009). Afterwards, the EEG data were segmented into epochs from −200 to 3000 ms relative to the onset of the static face, and a baseline correction was applied (−200 to 0 ms). Artifact‐afflicted epochs were removed using an amplitude threshold criterion of four standard deviations. ERPs were computed for each group (CI, NH), timepoint (pre‐ and post‐implantation), and condition (Target, NonTarget).

#### 2.4.3 VEP Analysis: Sensor Level

The analysis of the sensor‐level VEPs was divided into two steps. In a first step, we analyzed the VEPs in response to the onset of the static face (NonTarget, Target face). In a second step, we focused on the VEPs in response to the onset of the lip movement (NonTarget, Target word).

Regarding the first step, we analyzed the P1 and P2 VEPs in response to the onset of the static face. This was done by using an occipital region of interest (ROI) which included the electrodes PO3, POz, PO4, O1, Oz, and O2 (see Figure 3). A parietolateral ROI including the electrodes P7, PO7, P8, and PO8 was used for the face‐selective N170 component. Since the face categorization task was performed by means of an oddball paradigm, the P3b component was analyzed. Therefore, a parietal ROI including the electrodes P1, Pz, P2, PO3, POz, and PO4 was used for the P3b component following the onset of the static face for the Target face stimuli (face categorization task). For VEP quantification, individual peak amplitudes and latencies were measured by detecting the peak maximum and latency for each participant in commonly used latency bands of the P1, P2, N170, and P3b VEPs (Luck 2014; P1: 80–180 ms; N170: 160–180 ms, P2: 200–400 ms; P3b: 430–630 ms).

Regarding the second step, we used an explorative approach since the literature about VEPs to the lip movement onset is scarce. We computed a peak‐to‐peak measure, precisely the difference of the positive peak and the following negative peak in the time window between 600 and 800 ms (100–300 ms after the lip movement onset), which was compared between the groups (NH vs. CI), the conditions (attended vs. unattended words), and the timepoints (before vs. after implantation).

##### 2.4.3.1 Time‐Frequency Analysis

To get a deeper understanding of ongoing cortical face processing in CI users, we calculated event‐related spectral perturbations (ERSP) for each participant at each channel using a sinusoidal wavelet‐based analysis implemented in EEGLAB (Delorme and Makeig 2004). The number of cycles increased with frequency (start point 3‐cycle wavelet with sliding Hanning‐tapered window, 30 frequency steps from 1 to 30 Hz). Power changes across frequencies were baseline‐corrected by subtracting the mean baseline power spectrum (−200 to 0 ms). Similar to the more conventional VEP analysis (see last section), the ERSPs were averaged for an occipital ROI (PO3, POz, PO4, O1, Oz, and O2), and ERSP differences were computed between the separate conditions (attended vs. unattended words) within the groups. For statistical evaluation, two time‐frequency windows were defined, one after the onset of the static face (300–500 ms) and one after the onset of the lip movement (800–1100 ms), both in the frequency range of 8–18 Hz. The definition of those time‐frequency windows was based on the grand averages across all conditions and groups, so that they include the time‐frequency areas with the greatest values.

#### 2.4.4 VEP Analysis: Source Level

The Brainstorm software (Tadel et al. 2011) was used to compute cortical source activities following the tutorial of Stropahl et al. (2018). The software applies dynamic statistical parametric mapping (dSPM; Dale et al. 2000) to the data, which uses the minimum‐norm inverse maps with constrained dipole orientations to estimate the electrical activity of the neurons based on the scalp‐recorded measures. It localizes deeper sources more accurately compared to the standard minimum norm, but the spatial resolution is still blurred (Lin et al. 2006). Individual noise covariance matrices, and thereby individual noise standard deviations at each location, were calculated using the single‐trial pre‐stimulus baseline interval (−200 to 0 ms; Hansen et al. 2010). A standard three‐compartment boundary element head model was used as implemented in OpenMEEG (Gramfort et al. 2010; Stenroos et al. 2014). Based on the Destrieux‐Atlas (Destrieux et al. 2010), a visual and an auditory region of interest (ROI) were defined. The visual ROI consisted of one subregion to approximate the visual cortex (“S_calcarine”), whereas the auditory ROI consisted of three smaller regions to approximate Brodmann areas 41 and 42 (“G_temp_sup‐G_T_transv”, “G_temp_sup‐Plan_tempo”, “S_temporal_transverse”). Additionally, a subregion of the atlas was used to approximate the face area in the fusiform gyrus (“S_oc‐temp_med_and_Lingual”). Source activities were evaluated in these ROIs using the peak activation magnitude and latency for each individual participant. The activation data have absolute values and arbitrary units calculated by the normalization within the dSPM algorithm.

##### 2.4.4.1 Connectivity Analysis

To get measures of the connectivity between the used ROIs, a pre‐implemented procedure in Brainstorm was used (envelope correlation). In particular, a correlation is performed on the instantaneous amplitude, also called the envelope, of the analytic signal derived from the original data. This analytic signal is the result of a Morlet wavelet transformation. To reduce volume conduction and cross‐talk effects, the pairs of envelopes were orthogonalized prior to the connectivity computation (Hipp et al. 2012). The connectivity measure was calculated for the whole time of the stimulus (0–900 ms after stimulus onset).

#### 2.4.5 Statistical Analysis

The statistical analysis of the data was performed with the software R (Version 3.6.3, Vienna, Austria). The VEP data were separately examined on the sensor level (P1, N170, P2, and P3) and on the source level (visual, auditory, and fusiform ROI). Therefore, mixed‐model repeated‐measures ANOVAs with the between‐subject factor “group” (CI/NH) and the within‐subject factors “condition” (Target word/NonTarget) and timepoint (before implantation/after implantation) were computed for each analyzed component. Significant interactions and main effects (*p* < 0.05) were followed up by paired t‐tests and were corrected for multiple comparisons by the Holm‐Bonferroni approach (Holm 1979). In case of a violation of sphericity, a Greenhouse–Geisser correction was applied. To investigate the relationship between the measured VEPs and the speech perception with the CI, a correlation analysis was conducted using Pearson's correlation analysis.

## 3 Results

### 3.1 Behavioral Results

**FIGURE 2:**
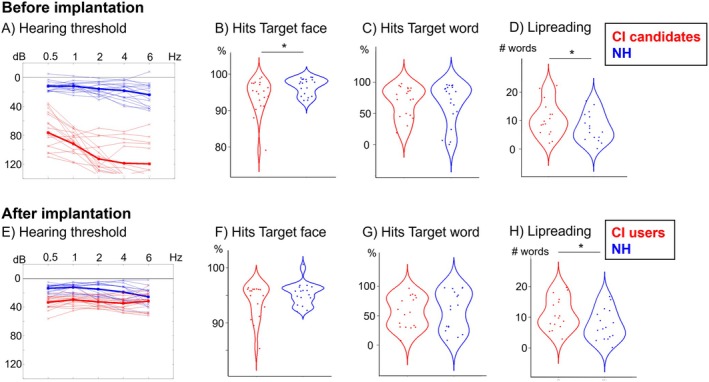
(A) Hearing threshold of all participants at the first timepoint (CI candidates before implantation). (B) Group‐specific hit rates for the Target face stimuli in the face categorization task at the first timepoint (CI candidates in red, NH in blue). (C) Group‐specific hit rates for the Target word stimuli in the word identification task at the first timepoint. (D) The number of correctly identified words in the additional behavioral lipreading task with bisyllabic words at the first timepoint. (E) Hearing threshold of all participants at the second timepoint (CI users after implantation). (F) Group‐specific hit rates for the Target face stimuli in the face categorization task at the second timepoint (CI users in red, NH in blue). (G) Group‐specific hit rates for the Target word stimuli in the word identification task at the second timepoint. (H) The number of correctly identified words in the additional behavioral lipreading task with bisyllabic words at the second timepoint. Asterisks indicate significant effects (p < 0.05).

Figure 2A,E show the hearing threshold of the participants, illustrating the regained hearing ability of the deafened participants after the CI switch‐on. The NH controls maintained a normal hearing ability throughout the study.

Regarding the two EEG paradigms, all participants showed hit rates of ≥ 79% in the face categorization task and ≥ 72% in the word identification task. This indicates that all participants were able to perform both tasks properly. The mean reaction times (RTs) ranged from 697 to 1250 ms (mean: 1025 ± 129 ms) in the face categorization task and from 1413 to 1963 ms (mean: 1703 ± 126 ms) in the word identification task. T‐tests between the groups (CI candidates/CI users vs. NH participants) showed for the *face categorization task* significantly better hit rates in the NH participants (*t*(29.13) = −2.08, *p* = 0.04) than in the CI candidates (Figure 2B). This group difference was observed specifically for the time before but not after implantation (Figure 2B). However, the performance in the *word identification task* and the response times were not different between the two groups at any timepoint.

Regarding the *additional behavioral lipreading task* (with natural speakers), the number of correctly reported words was compared between the groups at each timepoint (Figure 2D,H). This comparison revealed significantly better lipreading ability in CI users at both timepoints (before implantation (Figure 2D): *t*(29.25) = 2.27, *p* = 0.03; after implantation (Figure 2H): t(31.12) = 2.79, *p* = 0.01).

### 3.2 VEP Results: Sensor Level

#### 3.2.1 Cortical Processing of Static and Articulating Faces

**FIGURE 3:**
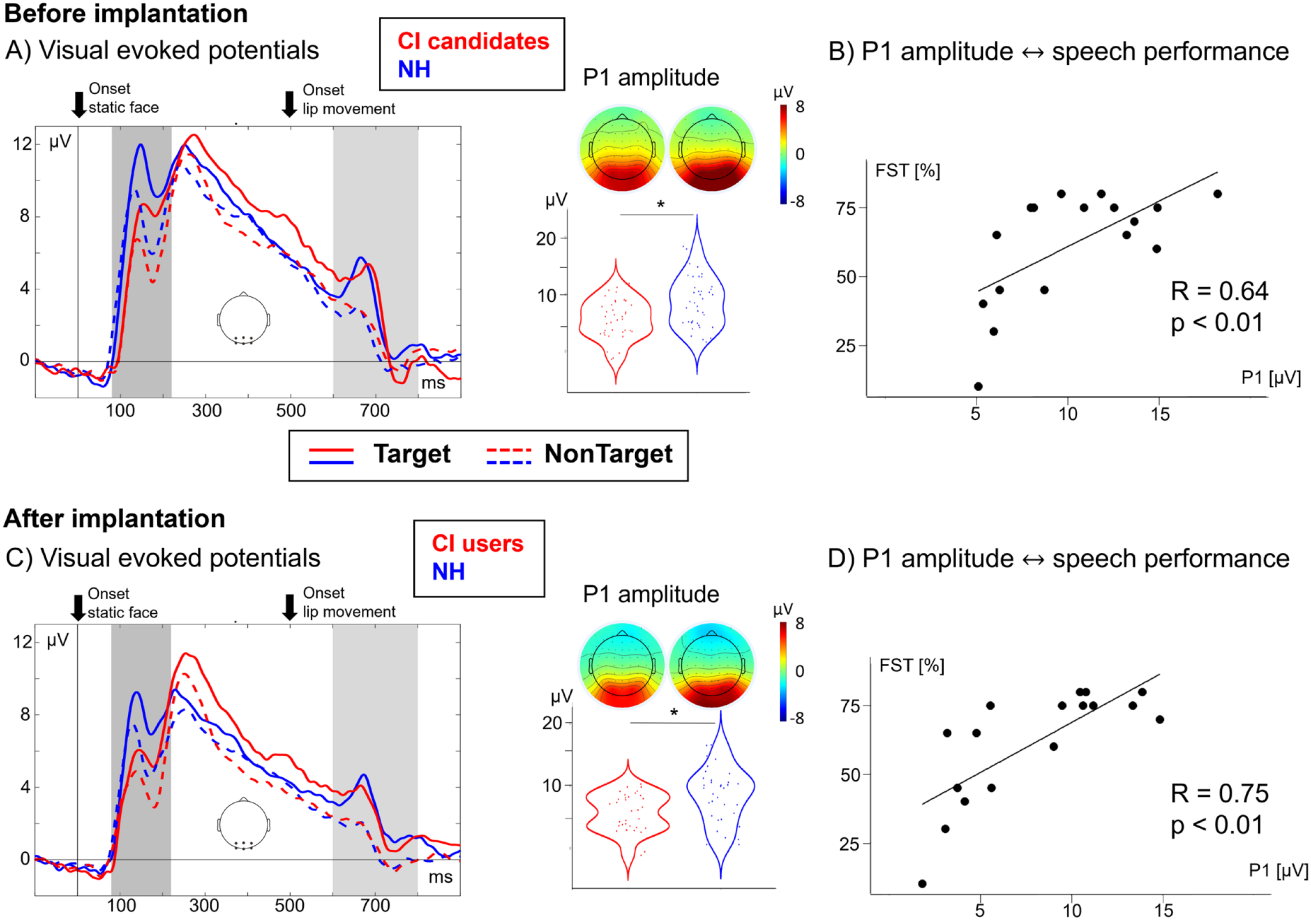
(A) VEPs over an occipital ROI showing a decreased P1 amplitude after the onset of the static face for the CI users before implantation when compared to NH listeners. Solid lines show the VEPs for the Target word stimuli (attended; word identification task), dashed lines show the VEPs for the NonTarget stimuli (unattended; face categorization task). The darker gray area (80–220 ms after stimulus onset) marks the time range for the P1 VEP following the onset of the static face, and the lighter gray area (600–800 ms after stimulus onset) marks the time range for the analysis of the VEP following the onset of the lip movement. Note that the difference between Targets and NonTargets reflect an attention effect, as the physically identical articulated words were processed attentively only in the Target word condition but not in the NonTarget condition. Boxplot showing the group difference for the P1 amplitude (averaged over both conditions) and topographies for both groups in the time range for the P1 (darker gray area). (B) Positive correlation between the P1 amplitude (face‐onset response) prior to implantation and the speech recognition ability after 6 months of CI use. (C) VEPs over an occipital ROI showing a decreased P1 amplitude after the onset of the static face for the CI users after implantation when compared to NH listeners. Boxplot showing the group difference for the P1 amplitude (averaged over both conditions) and topographies for both groups in the time range for the P1 (darker gray area). (D) Positive correlation between the P1 amplitude (face‐onset response) after implantation and the speech recognition ability after 6 months of CI use.

Figure 3 shows the grand average of VEPs in response to faces articulating Target words (i.e., attended processing of articulated words in the word identification task) and VEPs in response to faces articulating NonTargets (i.e., unattended processing of the same articulated words in the face categorization task) for both groups and timepoints in the occipital ROI. For all groups and conditions, the plots show pronounced VEP peaks in response to the face onset around 130 ms (P1) and 250 ms (P2), respectively (Figure 3A,D). In addition, there is a second, albeit smaller positive deflection around 660 ms in response to the onset of the lip movement (Figure 3A,D).

To address the question of whether there is a difference in the cortical processing of faces and lip movements between postlingually deafened subjects and NH listeners (Question 1), we compared the VEP peaks of the Target stimuli between the groups. Specifically, for the *P1 VEP face‐onset response*, we computed 2 × 2 × 2 mixed‐model repeated‐measures ANOVAs with the between‐subjects factor group (CI patients, NH participants) and the within‐subjects factor condition (Target face, NonTarget) and timepoint (before implantation, 6 months after implantation), separately for amplitudes and latencies. For P1 peak amplitudes, we found a main effect of group (*F*_1,31_ = 4.65, *p* = 0.04, η^2^ = 0.13), revealing a greater amplitude for the NH participants compared to the CI candidates/CI users independent of the condition and the timepoint. The *face‐selective N170 component*, occurring around 170 ms after the onset of the static face, was also evaluated by using similar 2 × 2 × 2 mixed ANOVAs. No effect of group was found, but a main effect of condition for the amplitude (*F*_1,31_ = 83.03, *p* < 0.01, η^2^ = 0.04) resulted in a greater N170 amplitude for the Target face stimuli when compared to the NonTargets, regardless of group or timepoint. Neither the P1 latency nor the N170 latency revealed any significant effects. Regarding the *P3 VEP* amplitude and latency, which were analyzed by a 2 × 2 mixed ANOVA with the within‐subjects factor timepoint (before implantation, 6 months after implantation) and the between‐subjects factor group (CI patients and NH participants), we did not observe any significant effects.

A correlation analysis between the P1 amplitude (recorded at the time before and after implantation) and the Freiburg monosyllabic word test (performed at the time with 6 months of CI experience) resulted in a positive correlation at both time points (P1 before implantation (Figure 3B): *R* = 0.64, *p* < 0.01; P1 after implantation (Figure 3E): *R* = 0.75; *p* < 0.01), showing that a greater response to the static face results in a better speech recognition ability after cochlear implantation.

#### 3.2.2 Attention Effect on VEPs

To investigate the influence of attention (attended Target words versus unattended NonTargets) on VEPs elicited by articulating faces (Question 2), we computed a 2 × 2 × 2 ANOVA on the peak‐to‐peak amplitude for the time window after the lip movement. This ANOVA included the between‐subjects factor group (CI candidates/users, NH participants) and the within‐subjects factors condition (Target word, NonTarget) and timepoint (before implantation, 6 months after implantation). The results revealed a main effect of condition (*F*_1,32_ = 41.60, *p* < 0.01, η^2^ = 0.18), indicating a larger peak‐to‐peak amplitude for the Target word condition (i.e., attended processing of articulated words in the word identification task) than for the NonTarget condition (i.e., unattended processing of the same articulated words in the face categorization task), independent of the group and the timepoint.

**FIGURE 4:**
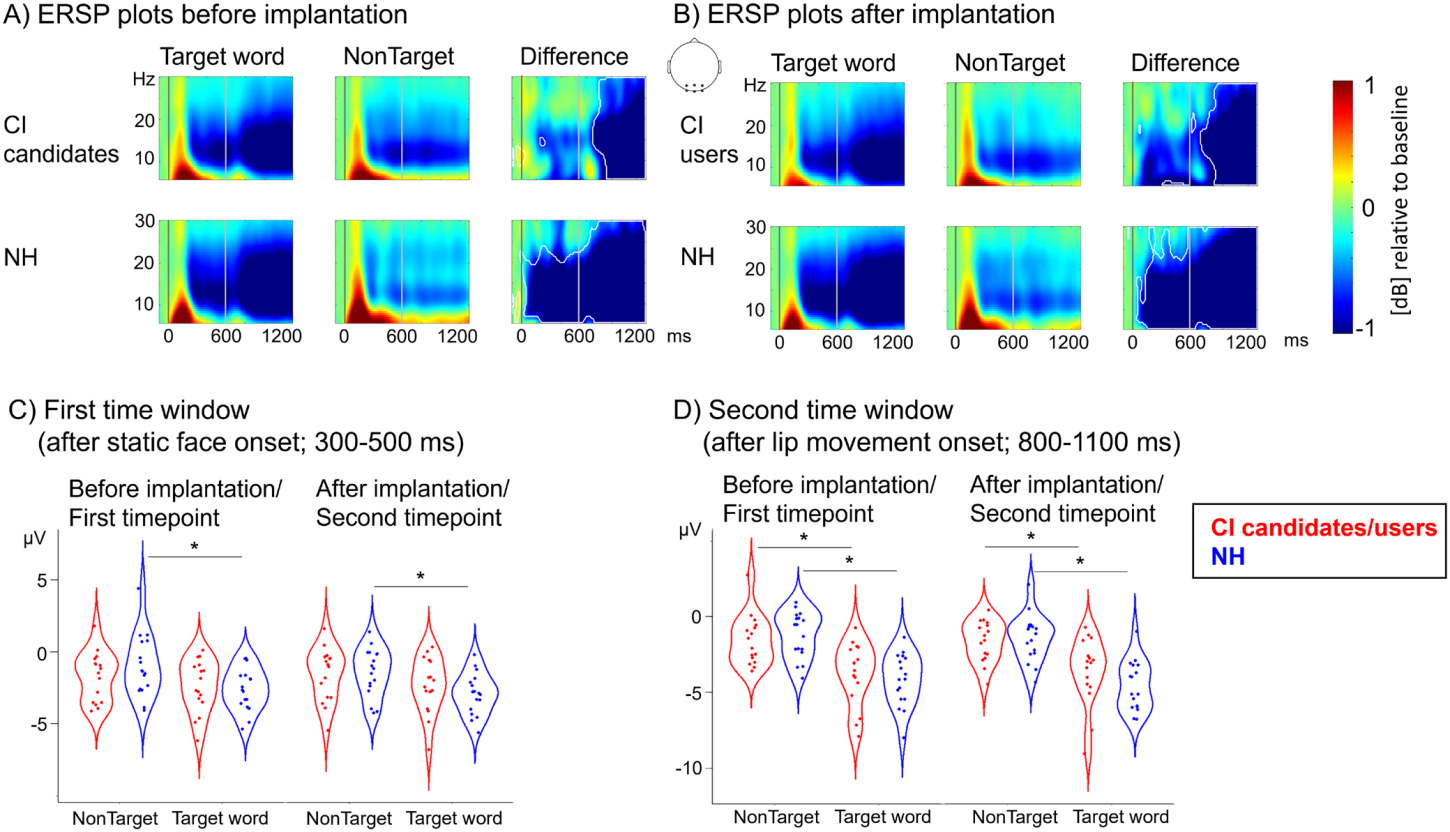
(A) Event‐related spectral perturbation (ERSP) plots for the time before (A) and after (B) implantation. The darker gray line represents the onset of the static face (0 ms), while the lighter gray line represents the onset of the lip movement (600 ms) in each plot. The analyses were conducted in two time windows, in particular one after the onset of the static face (0–300 ms = first time‐frequency‐window) and one after the onset of the lip movement (650–800 ms = second time‐frequency‐window). The conditions Target word (word identification task) and NonTarget (face categorization task) are shown separately (first and second column), as well as the difference between these two conditions within each group (third column). Note that significant within‐group differences reflect an attention effect, as the physically identical articulated words were processed attentively only in the Target word condition but not in the NonTarget condition. (C) Boxplots of mean power values (8–18 Hz) in the first time‐frequency‐window (after the onset of the static face) for the CI group in red and the NH group in blue for both timepoints (before/after implantation). The asterisks indicate significant differences (*p* < 0.05). Note that the NH listeners but not the CI candidates/CI users showed a significantly decreased power in the 8–18 Hz frequency range (i.e., significantly more negative values) for the Target condition compared to the NonTarget condition. (D) Boxplots of mean power values (8–18 Hz) in the second time‐frequency‐window (after the onset of the lip movement) for the CI group in red and the NH group in blue for both timepoints (before/after implantation). The asterisks indicate significant differences (*p* < 0.05). Note that both groups showed a significantly decreased power in the 8–18 Hz frequency range (i.e., significantly more negative values) for the Target condition compared to the NonTarget condition.

Additionally, we performed a time‐frequency analysis by means of event‐related spectral perturbations (ERSP). Figure 4 shows the time‐frequency plots for the Target words (attended processing in the word identification task) and the NonTargets (unattended processing in the face categorization task) for both groups and timepoints (Figure 4A,B). Further, the figure illustrates the condition differences for both groups in the occipital ROI. All conditions show an increase in power for both groups *after the onset of the static face (first time window; 0–300 ms)*, especially in the lower frequencies (≤ 10 Hz). This is followed by a time window with negative values starting around 300 ms after the onset of the static face in the frequency range of 8–18 Hz. Although both groups show these negative values in both the Target word condition and the NonTarget condition, they are mostly pronounced in NH listeners in the Target word condition (i.e., most negative power values). A second time window of enhanced power in the low frequencies (≤ 7 Hz) can be seen *after the onset of the lip movement (second time window; 650–800 ms)* in both groups, especially for the Target word condition. This power increase is again followed by a time window with negative values in the 8–18 Hz range. Since it was not possible to use a longer baseline for the time‐frequency analysis, the results about alpha power should be interpreted with caution.

The statistical analysis, realized using a 2 × 2 × 2 ANOVA with the between‐subjects factor group (CI candidates/users, NH participants) and the within‐subjects factors condition (Target word, NonTarget) and timepoint (before implantation, 6 months after implantation) for each predefined time‐frequency window, revealed an interaction between group and condition (*F*_1,32_ = 4.22, *p* = 0.05, η^2^ = 0.02) for the *time window following the static face onset*. Post hoc tests showed specifically for the NH listeners but not for the CI group a significantly decreased power (i.e., more negative power values) in the 8–18 Hz frequency range for the Target condition compared to the NonTarget condition (*p* < 0.01). For the *time window after the lip movement onset*, both groups showed decreased power (i.e., more negative power values) for the Target word condition when compared to the NonTarget condition (NH: *p* < 0.01, CI: *p* < 0.01). We did not find a significant effect of the factor timepoint on the ERSP.

### 3.3 VEP Results: Source Level

#### 3.3.1 Cortical Processing of Static and Articulating Faces

**FIGURE 5:**
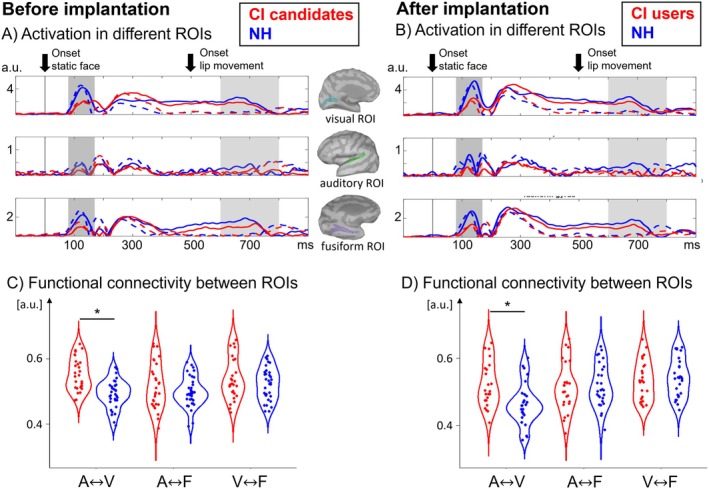
(A) Mean activities for the visual ROI, the auditory ROI, and the fusiform gyrus for both groups at the first timepoint. (B) Mean activities for the visual ROI, the auditory ROI, and the fusiform gyrus for both groups at the second timepoint. The plot shows a decreased activation following the face onset at P1 latency range for the CI candidates and CI users when compared to the NH listeners. (C) Mean functional connectivity, as indicated by envelope correlation values (0–900 ms after stimulus onset), for all combinations of the three ROIs at the first timepoint for the attended condition: Auditory ROI (A) vs. fusiform gyrus (F), auditory ROI (A) vs. visual ROI (V), visual ROI (V) vs. fusiform gyrus. (D) Mean functional connectivity, as indicated by envelope correlation values (0–900 ms after static face onset), for all combinations of the three ROIs at the second timepoint for the attended condition. The plot shows enhanced functional connectivity between the visual and auditory ROI for the CI candidates and the CI users when compared to the NH listeners.

Figure 5 illustrates the activation in the visual ROI, the auditory ROI, and the fusiform gyrus for the Target word (word identification task) and the NonTarget stimuli (face categorization task) for both groups and timepoints. The activation in the different ROIs showed deflections to the onset of static faces and lip movements with latencies that are comparable to the sensor level. A 2 × 2 × 2 ANOVA with the between‐subjects factor group (CI patients, NH participants) and the within‐subjects factors condition (Target word, NonTarget), and timepoint (before implantation, 6 months after implantation) was computed for the peak activation magnitude and latency within each ROI. The results revealed a main effect of group in the visual ROI for the face‐onset response at P1 latency range (*F*_1,32_ = 4.21, *p* = 0.05, η^2^ = 0.06). Similar to the observations at the sensor level, the NH listeners showed greater responses when compared to the CI patients. Additionally, a main effect of condition was found for the face‐onset response at P1 latency range in the fusiform gyrus (*F*_1,32_ = 5.83, *p* = 0.02, η^2^ = 0.02), showing a greater activation in response to the Target word condition when compared with the response to the NonTarget condition. Regarding the cortical activation following the onset of *lip movements*, a main effect of condition was found in the visual ROI (*F*_1,32_ = 16.05, *p* < 0.01, η^2^ = 0.14) and the fusiform gyrus (*F*_1,32_ = 11.57, *p* < 0.01, η^2^ = 0.11), with the result of greater activation for the Target word compared to the NonTarget condition. Although the average waveform of NH listeners in the visual ROI indicates some variability, the test–retest reliability of the peak measures between the two timepoints was very high for this group of participants (*r* = 0.88).

#### 3.3.2 Connectivity

The analysis of the functional connectivity (Figure 5C,D) was done by a 2 × 2 × 3 ANOVA with the between‐subjects factor group (CI patients, NH participants) and the within‐subject factor timepoint (before implantation, 6 months after implantation) and direction (auditory ROI vs. visual ROI, auditory ROI vs. fusiform gyrus, visual ROI vs. fusiform gyrus) for the Target word condition. The results revealed a greater connectivity between the auditory and visual ROI for the CI candidates/CI users compared to the NH participants (*F*_1,32_ = 9.95, *p* = 0.05, η^2^ = 0.14). No significant group differences were found for the connectivity between the fusiform gyrus on the one hand and the auditory or visual ROI on the other hand.

## 4 Discussion

The present prospective longitudinal study compared visual‐evoked potentials (VEPs) to static and articulating faces between NH listeners and postlingually deafened individuals before and after cochlear implantation. As far as we are aware, this is the first study to examine visual face processing in attended and unattended conditions before and after cochlear implantation within the same individuals. Regarding our first research question of whether postlingually deafened individuals and NH listeners differ in the cortical processing of static and articulating faces (Question 1), the results revealed a reduced cortical response in the visual cortex at P1 latency and an enhanced functional connectivity between the visual and the auditory cortex for the CI candidates when compared to the NH controls. Comparing the groups in terms of attention effects (processing of attended vs. unattended faces; Question 2), the event‐related spectral perturbations (ERSP) revealed a group‐specific pattern particularly in the (extended) alpha frequency range (8–18 Hz). The NH listeners but not the CI candidates showed a pronounced power increase (i.e., less negative power values) for unattended than attended faces. The alterations in cortical face processing of CI candidates remained largely unchanged after 6 months of CI experience (Question 3). Nevertheless, we observed a positive correlation between the (reduced) visual P1 amplitude *before* implantation and the speech recognition ability *after* implantation, pointing to a link between cortical visual reorganization and CI outcome (Question 4).

### 4.1 Behavioral Data

Sensory deprivation can have an impact on the remaining senses. Two explanations of this impact have been proposed in the literature. The so‐called *perceptual deficit hypothesis* states that a deficit in one sensory modality can affect the organization and development of other sensory systems (e.g., Myklebust 1964), whereas the *sensory compensation hypothesis* states that a deficit in one sensory modality can be compensated for by making the other modalities more sensitive (e.g., Gibson 1969). Both hypotheses have been supported by studies in hearing‐impaired individuals. Some studies revealed deficient visual abilities in congenitally deaf compared to hearing individuals (e.g., Quittner et al. 2004; Parasnis et al. 2003), while others found supranormal abilities for deaf individuals (e.g., Bottari et al. 2011; Hong Lore and Song 1991; Neville and Lawson 1987). A third group of results even indicated comparable visual performance between deaf and hearing individuals (e.g., Parasnis 1983; Bavelier et al. 2006; Mitchell and Maslin 2007). These variations in results underscore the ongoing debate of perceptual and cognitive functions in sensory deprived individuals. Part of the heterogeneity can be explained by different groups of participants (e.g., native signers/non‐signers), the characteristics of the stimuli, the eccentricity of the visual stimuli (periphery/focus), and the task conditions (bottom‐up/top‐down). Consistent with previous results about congenitally deaf individuals (Bottari et al. 2014; Finney et al. 2003; Hauthal et al. 2014), we found an enhanced lipreading ability in the postlingually deafened CI group not only before implantation but also 6 months after implantation (Rouger et al. 2007; Layer et al. 2022) as compared to NH subjects. These findings highlight that hearing‐impaired individuals rely on visual cues to understand speech and that this visual compensation does not decrease after cochlear implantation. The fact that the group difference was only found in the additional behavioral lipreading task (including a list of 21 monosyllabic words frequently used in the clinical context), but not in the EEG word identification task, can be explained by a ceiling effect in the latter. Although the NH listeners from this study were poorer lipreaders than the CI users, they were accustomed to the three articulated words in the EEG word identification task, resulting in high performance levels. Similarly, previous EEG studies have reported comparable performance between CI users and NH subjects for syllables in conditions with low task difficulty (Layer et al. 2022; Stropahl and Debener 2017). Hence, our results confirm previous conclusions that behavioral visual improvements in hearing‐impaired individuals and CI users are stimulus‐ and task‐selective and that these improvements are especially pronounced in difficult conditions with complex speech stimuli (Rouger et al. 2008; Tremblay et al. 2010; Hauthal et al. 2013).

In contrast to former studies (e.g., Bottari et al. 2011), we did not find enhanced visual reactivity for the deafened participants before and after implantation. This can be explained by the heterogeneity of the visual characteristics of the stimuli and the diversity in the sample characteristics across studies. Indeed, in the current study, we presented the stimuli in the center of the screen, whereas most of the previous studies reported faster visual reactivity in deaf individuals when stimuli were presented in the peripheral visual field (e.g., Bavelier et al. 2000).

In conclusion, our behavioral results support the *sensory compensation hypothesis* since our auditory‐deprived patients (before and after implantation) showed enhanced visual lipreading ability, particularly in difficult task conditions.

### 4.2 Cortical Reorganization Following Sensory Deprivation and Cochlear Implantation

#### 4.2.1 Intra‐Modal Reorganization in the Visual Cortex Before and After Implantation

The present results show a decreased scalp‐recorded visual P1 amplitude following the onset of a static face for the CI group compared to NH listener*s*. This observation was confirmed by the source analysis, which revealed a decreased amplitude in the visual cortex of deafened participants (before and after implantation) when compared to the NH subjects. Basically, a smaller VEP can be explained either by a smaller congregation or by reduced synchronization of the activated neurons in the deafened individuals' visual cortex (Nunez, 1981); however, the applied source analysis does not distinguish between these two mechanisms. In conclusion, we found evidence for intra‐modal reorganization in the visual cortex of postlingually deafened individuals before implantation, which was previously reported for early deaf individuals (Bavelier et al. 2000; Bottari et al. 2014; Hauthal et al. 2013) and postlingually deafened CI users after implantation (Sandmann et al. 2012). In early deaf individuals, Bottari et al. (2014) found a reduced P1 response in *visual cortical areas* for visual stimulation of the central visual field when compared to NH listeners. By contrast, Bavelier et al. (2000) reported enhanced activation in the *motion‐selective area MT/MST* in deaf individuals, especially for moving visual stimuli in the periphery. Although previous results (with visual stimulation in the central visual field) have suggested that a reduced response in the visual cortex is paired with a recruitment of the auditory cortex (Bottari et al. 2014; Sandmann et al. 2012), the present results did not show significant group differences in the auditory cortex and the fusiform gyrus. This discrepancy in results may be explained by differences in stimuli (basic vs. more complex) and experimental tasks (detection vs. categorization and lipreading). Importantly, however, our CI users showed functional connectivity that was specifically enhanced between the visual and auditory cortex, both before and after implantation. This points to stronger interactions between the visual and auditory cortex that are induced by auditory deprivation and that persist even after 6 months of CI experience (see section 4.2.2 for a more detailed discussion of the functional connectivity). Thus, our results obtained with static and articulating face stimuli only partially support previous results. Although they cannot confirm a pronounced cross‐modal activation of auditory cortex for static and articulating faces, they are in line with previous reports of enhanced audio‐visual interactions in CI users (e.g., Chen et al. 2017; Schierholz et al. 2015).

Intra‐modal changes in visual cortical areas have been reported not only in congenitally deaf individuals but also in CI users (Giraud et al. 2001; Strelnikov et al. 2010). Indeed, Sandmann et al. (2012) also found a decreased visual cortical response at P1 latency range in response to simple, reversing chequerboard patterns. Since Sandmann et al. (2012) performed a cross‐sectional study, they could not conclude whether the changed visual processing was linked to the period of deafness or to the adaptation to the new auditory input via the CI. However, the current longitudinal study shows that a deprivation‐induced alteration in the processing of static and articulating faces hardly changes over the first six months of CI use. Thus, the visual intra‐modal cortical alterations observed in CI users seem to be mainly induced by auditory deprivation and not by CI experience.

#### 4.2.2 Cross‐Modal Reorganization and Audio‐Visual Connectivity Before and After Implantation

Although we could not replicate previous observations of pronounced cross‐modal activation in the auditory cortex of CI users, our results confirm previous reports of greater functional connectivity between the visual and auditory cortex for attended visual word processing in postlingually deafened individuals compared to NH controls (e.g., Strelnikov et al. 2015; Chen et al. 2017). This indicates a stronger cross‐modal association which may help the hearing‐impaired individuals to compensate for the missing (before implantation) or limited (after implantation) auditory input (Schierholz et al. 2015). The underlying mechanism of stronger functional connectivity could indicate an enhancement of pre‐existing connections between the visual and auditory cortices (Merabet and Pascual‐Leone 2010). This is consistent with previous studies with congenitally deaf animals and humans which found increased structural and functional connectivity between the visual and auditory cortex (Kok et al. 2014; Kral 2007). Moreover, Lazard and Giraud (2017) found a right hemispheric phonological network with participation of the visual cortices, predicting poor CI outcome. They used a rhyme decision task and fMRI measures to capture functional connectivity. However, MRI scans are difficult to perform with CI users because the implants overshadow large areas of the brain (Majdani et al. 2009), making it difficult to measure connectivity with the auditory cortex after implantation. By contrast, the present study used EEG measures which allowed us to study the functional connectivity between the visual and auditory cortex in CI users both at the time points before and after implantation. Given that we found comparably strong audio‐visual connectivity at the two timepoints, our results suggest that this cross‐modal enhancement is mainly induced by auditory deprivation and not by CI experience. Nevertheless, future research is needed to understand the pinpoint mechanisms more precisely. Furthermore, because our study used face stimuli, while others used simpler stimuli like chequerboards (Chen et al. 2017) or written words (Lazard and Giraud 2017), stimulus dependence should be considered in future studies.

### 4.3 Deaf People's Attention to Visual Lip Movements Is Enhanced

To date, the cortical processing of lip movements has been mostly discussed in conjunction with simultaneous auditory input (Stropahl et al. 2017; Layer et al. 2022). A recent study by Paul et al. (2022) used purely visual stimuli of a male speaker articulating monosyllabic words and analyzed the P1 and N1 VEP components following the onset of the video. Since the onset of the video was also the onset of the lip movement, they were not able to differentiate the processing between static faces and lip movements. Interestingly, they did not find a difference between CI users and NH listeners for P1 and N1 VEPs. The current study extends these previous findings by examining the face and word processing systematically at different stages in purely visual conditions. In particular, we analyzed the evoked responses not only to the static face onset but also to the onset of the lip movement. As shown in Figure 3A,D, we found a larger VEP amplitude for the attended lip movement when compared to the unattended lip movement in both groups, which reflects an effect of attention in both groups (Gazzaley et al. 2008). In contrast to the ERSP results, which revealed a group‐specific pattern particularly in the frequency range 8–18 Hz (see 4. Discussion in the next section), we did not find a significant group difference in the attention effect for the VEP amplitude (peak detection analysis) at both time points. This discrepancy in findings for different approaches supports the view that a time‐frequency analysis allows a more differentiated insight into cortical processes than the more conventional VEP peak detection analysis.

Rhythmic activity in the alpha frequency band has been related to the expectation and allocation of attention to attended stimuli (Foxe and Snyder 2011), where increased alpha power (i.e., less negative power values) is proposed to indicate the suppression of unattended stimulus features (Foxe and Snyder 2011; Klimesch et al. 2007; Jensen and Mazaheri 2010). In the presented results, the NH group shows a *decreased* oscillatory activity in the 8–18 Hz frequency band (i.e., enclosing the alpha frequency band) after face onset and prior to lip movement onset for the attended Target word stimuli compared to the ignored NonTarget word stimuli. This relative *suppression* of alpha power prior to the onset of the *lip movement* might indicate the allocation of attention away from the irrelevant NonTarget stimuli (in the face‐categorization task) and towards the relevant Target stimuli (in the word‐identification task). Consequently, the *suppression* of alpha power prior to lip movement in NH listeners can be interpreted as a *facilitation* of relevant information processing in the Target word condition. By contrast, no such significant differences between Target and NonTarget stimuli were observed in the CI group (Figure 4A,B) before lip movement onset. CI candidates and, after implantation, CI users heavily rely on visual information due to the limited auditory input. Therefore, one might speculate that CI candidates/users would not deliberately withdraw processing resources from the visual modality to facilitate processing in other modalities (Foxe et al. 1998). Indeed, in experienced CI users, a more pronounced weighting of visual attention has been suggested previously to compensate for the (still limited) electrical hearing (Butera et al. 2018; Radecke et al. 2022). Also, in deaf individuals, a more distributed visual attention has been observed (Bottari et al. 2010), which further confirms the view of a modulated visual attention in hearing‐impaired individuals. Similar mechanisms might have prevented the suppression or withdrawal of attention from task‐irrelevant (unattended) visual face stimuli before lip movement onset in our CI candidates/users of this study. However, as soon as visually relevant information is present (i.e., starting with the lip movement onset), differences in 8–18 Hz power between Target and NonTarget stimuli might indicate the allocation of attention towards the relevant lip movements and a suppression of irrelevant face features in both NH and CI groups. This can be seen in the significant condition differences for both groups at both timepoints.

### 4.4 Face Selective N170 Component

Faces have been shown to be perceived in a different way than other abstract visual objects (Stropahl et al. 2017). A core region in the neural network for face processing is the fusiform face area (Haxby et al. 2000; Kanwisher et al. 1997; Kanwisher and Yovel 2006). In electrophysiological data, face selectivity is most prominent around 170 ms after face onset over occipito‐temporal scalp regions (N170 component; Bentin et al. 1996; Bötzel and Grüsser 1989; Rossion and Jacques 2008). Some previous studies showed advantageous face processing for deaf compared to NH individuals, as evidenced by greater amplitudes of the N170 component for deaf individuals. This is explained by the compensation of missing auditory input during face‐to‐face communication (Kral 2013; Mitchell et al. 2013; Woodhouse et al. 2009; Stropahl et al. 2015). The present results do not replicate these previous findings, neither for (postlingually deafened) CI candidates before implantation nor for CI users after implantation. This lack of a group difference may be explained by the fact that we used computer‐animated faces instead of real faces and that in our paradigms, there were similar faces rather than a variation with other objects, such as houses (Stropahl et al. 2017). However, we observed that in the *face categorization task*, the N170 amplitude was enhanced for rare deviant faces when compared to the more frequent NonTarget word stimuli. This effect was observed across both groups and might be explained by the structure of the paradigm and cortical adaptation: In the face categorization task, the *frequent* NonTarget words were *not task‐relevant*, but the *rare* deviant Target face stimuli were *task‐relevant*. Thus, our results indicate that the N170 response is enhanced in conditions with rare and task‐relevant stimuli, regardless of whether the individuals have normal hearing or have a hearing loss.

### 4.5 Relationship Between Cortical Markers and Speech Understanding via the CI

Several previous studies have focused on the relationship between cortical plasticity and the speech recognition ability via a CI. While cross‐modal plasticity is conceived as adaptive for behavior in the remaining senses after sensory loss (e.g., Amedi et al. 2003, Gougoux et al. 2005; see for a review Voss et al. 2010), it has been debated whether it is maladaptive for sensory restoration (e.g., Doucet et al. 2006; Rouger et al. 2012; Sandmann et al. 2012; Paul et al. 2022). Rouger et al. (2012) stated that auditory, visual, and audiovisual networks are involved in cortical changes after cochlear implantation and that audiovisual integration after cochlear implantation is supported by functional links between face and voice processing areas. We found no evidence for enhanced cross‐modal activation in the auditory cortex of CI candidates/CI users, but we did find alterations in functional connectivity between the auditory and visual cortex in these individuals (see Figure 5C,D). Because the connectivity values do not correlate with speech recognition ability via the CI, we cannot contribute to the discussion about the adaptive or maladaptive effects of cross‐modal plasticity based on our findings. However, our results show a positive correlation between the (reduced) scalp‐recorded visual P1 amplitude before and after implantation on the one hand, and the speech recognition ability after 6 months of CI use on the other hand. This indicates that less deprivation‐induced (intra‐modal) cortical reorganization within the visual cortex (as indicated by a larger, i.e., closer to the NH listeners' P1 amplitude) relates to a better CI outcome. Thus, the cortical marker P1 amplitude—reflecting the extent of cortical reorganization—may be one out of several factors that can help explain the large variability in CI outcome. Indeed, Strelnikov et al. (2013) found a correlation between visual cortex activation and auditory speech perception in postlingually deafened CI users, indicating a synergy between both modalities. However, future studies with a larger sample size need to verify whether the cortical P1 VEP amplitude can help give a better prognosis of the expected CI outcome at the time before implantation. Since our prospective longitudinal study was restricted to 17 patients, this result should be interpreted with caution and future research with larger cohorts is needed to validate this finding.

## 5 Conclusions

This prospective longitudinal study examined the cortical processing of static and articulating faces in attended and unattended conditions. We observed that CI users before implantation show enhanced lipreading ability and deprivation‐induced changes in the cortical processing of static and articulating faces (Question 1). Specifically, these alterations emerge not only in functional changes within the visual cortex (as indicated by a reduced face‐onset response at P1 latency) but also in an enhanced functional connectivity between the visual and auditory cortex at sensory processing stages. The same CI candidates (before implantation) also show altered allocation of attention to static and articulating faces when compared to NH listeners, as indicated by more comparable alpha power in the response to task‐relevant and task‐irrelevant faces (Question 2). This finding suggests that CI candidates are highly attentive to lip movements, regardless of whether these visual stimuli are task‐relevant or task‐irrelevant. Even after restoration of the auditory input via the CI, the (reduced) face‐onset response in the visual cortex and the (enhanced) functional connectivity between the visual and auditory cortex remain largely unchanged, as well as the reduced differentiation between relevant and irrelevant visual information (Question 3). In sum, these results suggest pronounced, deprivation‐induced cortical alterations in the processing of static and articulating faces, which appear to be hardly changed over the first 6 months of CI usage. Our observation that the scalp‐recorded P1 amplitude, as measured before implantation, was positively related to the speech recognition ability after implantation (Question 4) suggests a connection between the deprivation‐induced cortical reorganization and the CI outcome. Thus, the P1 amplitude, which might indicate the extent of intra‐modal reorganization of the visual cortex, may provide valuable information for the prognosis of the CI outcome.

## Author Contributions

A.W. performed and analysed all experiments, put together all figures and the manuscript draft. N.L. helped with experiments and analysing data and reviewed the manuscript. J.‐O. R. helped with methodology and data analysis and reviewed the manuscript. H.M. was involved in funding acquisition, provided important software, and reviewed the manuscript. V.M. helped with patient recruitment and reviewed the manuscript. R. L.‐R. and M.W. were involved in conceptualisation of the study and reviewed the manuscript. P.S. was the supervisor of the study, had a central role in the conceptualisation, acquired the funding and helped writing the manuscript.

## Conflicts of Interest

The authors declare no conflicts of interest.

## Supporting information


Data S1.

## Data Availability

Raw data can be provided upon reasonable request to the corresponding author.
